# Alteration of stream temperature by natural and artificial beaver dams

**DOI:** 10.1371/journal.pone.0176313

**Published:** 2017-05-17

**Authors:** Nicholas Weber, Nicolaas Bouwes, Michael M. Pollock, Carol Volk, Joseph M. Wheaton, Gus Wathen, Jacob Wirtz, Chris E. Jordan

**Affiliations:** 1 Eco-Logical Research Inc., Providence, Utah, United States of America; 2 Watershed Sciences Department, Utah State University, Logan, Utah, United States of America; 3 Northwest Fisheries Science Center, Seattle, Washington, United States of America; 4 South Fork Research Inc., North Bend, Washington, United States of America; University of Minnesota, UNITED STATES

## Abstract

Beaver are an integral component of hydrologic, geomorphic, and biotic processes within North American stream systems, and their propensity to build dams alters stream and riparian structure and function to the benefit of many aquatic and terrestrial species. Recognizing this, beaver relocation efforts and/or application of structures designed to mimic the function of beaver dams are increasingly being utilized as effective and cost-efficient stream and riparian restoration approaches. Despite these verities, the notion that beaver dams negatively impact stream habitat remains common, specifically the assumption that beaver dams increase stream temperatures during summer to the detriment of sensitive biota such as salmonids. In this study, we tracked beaver dam distributions and monitored water temperature throughout 34 km of stream for an eight-year period between 2007 and 2014. During this time the number of natural beaver dams within the study area increased by an order of magnitude, and an additional 4 km of stream were subject to a restoration manipulation that included installing a high-density of Beaver Dam Analog (BDA) structures designed to mimic the function of natural beaver dams. Our observations reveal several mechanisms by which beaver dam development may influence stream temperature regimes; including longitudinal buffering of diel summer temperature extrema at the reach scale due to increased surface water storage, and creation of cool—water channel scale temperature refugia through enhanced groundwater—surface water connectivity. Our results suggest that creation of natural and/or artificial beaver dams could be used to mitigate the impact of human induced thermal degradation that may threaten sensitive species.

## Introduction

Temperature affects biological, physical, and chemical stream processes and has a profound influence on the structure and function of lotic systems [1,2]. Physiological thresholds and behavioral responses to temperature also govern the distribution of stream organisms and assembly of stream communities [3–5]. Stream temperature regimes are determined by interactions between internal characteristics of the channel, the riparian zone, the alluvial aquifer, and external environmental drivers such as climatic and catchment conditions that deliver water and thermal energy [6]. The realization that anthropogenic activities (e.g., irrigation withdrawals, channel straightening, riparian and upland vegetation loss) are resulting in widespread alteration of stream temperature regimes, coupled with the inevitable impacts of global climate change have intensified the need to understand the factors that influence stream temperature and develop approaches for mitigating the effects of temperature degradation [6–8].

Beaver (*Castor canadensis*) have long been recognized as ecosystem engineers [9,10] and their dam building and foraging behavior alters channel, riparian, and hydrologic processes in manners that can influence stream temperature dynamics [11,12]. Relying on woody riparian vegetation as their primary source of food and material for dam construction [13], the presence of beaver may result in the acute depletion of shade—providing vegetation and addition of radiant heat to surface water [14,15]. In addition, expansion of water surface area and reduced flow velocity in ponds may increase the susceptibility of surface water to radiant heating and thermal exchange with ambient air temperature through conduction, convection, and evaporation [14,15]. These conspicuous impacts (i.e., vegetation depletion, increased pond area) support the assumption that beaver dams increase water temperatures, and are often invoked as reasons why beaver may be detrimental to temperature sensitive biota such as salmonids [16].

However, research focusing on the impacts that beaver dams have on stream temperature support conflicting conclusions [17,18]. For example, several studies have reported increases in stream temperature downstream of beaver dams or dam complexes [19,20], an extreme example of which was reported by Margolis [21], where a 7°C increase in temperature was documented as headwater streams passed through large (~5 ha) beaver dam complexes. In contrast, research also supports little to no influence of beaver dams to stream temperatures [22], or report that beaver dams lead to a reduction or buffering of summer temperature extremes [12,23,24]. Increased water storage, hydraulic head, and deposition of alluvial material behind dams often results in an increase to the elevation of alluvial aquifers and rates of groundwater and surface water exchange [20]. Broader spatial distribution and an increased rate of cool—groundwater infiltration has the potential to increase surface water temperature heterogeneity, and to moderate extreme temperatures during periods of baseflow discharge [25].

Inconsistent findings concerning the thermal impacts of beaver dams may stem from research that encompasses a limited temporal (i.e., several days to single seasons) and/or spatial (i.e. single pond or complex) extent. Further, many studies have also been conducted without appropriate comparisons with unimpounded stream reaches that might effectively elucidate the effects that beaver impoundments have on stream temperature [18]. In addition, the magnitude by which beaver influence hydro-geomorphic stream processes that might affect temperature depend on variation among and throughout the lifespan of individual dams and dam complexes, as well as climatic and geologic characteristics of specific catchments. Understanding the real and perceived influence that beaver dams have on stream temperatures requires more longitudinal and geographically expansive studies, as well as research designs that provide stronger inference. The need for a deeper understanding of how beaver dams influence stream temperatures is amplified by the recent trend that promotes the use of beavers and structures designed to simulate the function of beaver dams as restoration tools. Advocates of these tools claim they can be used to enhance riparian and floodplain function, increase stream geomorphic complexity, mitigate for climate change, and increase salmonid productivity [26–28].

In this study, we sought to describe how development of beaver dam complexes may impact surface water temperature regimes. To this end, we tracked beaver dam distributions and continuously monitored stream temperatures at 22 locations throughout 34 km of stream channel over an eight-year period between 2007 and 2014. During the course of the study the number of natural beaver dams increased by an order of magnitude, and an additional 4 km of stream were subjected to a restoration manipulation that consisted of installing a high-density of Beaver Dam Analog (BDA) structures designed to mimic the function of natural beaver dams [29]. In addition, we investigated the potential for beaver complexes to contribute to small scale temperature heterogeneity by comparing temperature distributions throughout a beaver pond complex with that of an unimpounded stream reach. The specific goals of our research and analyses were to: 1) document the potential for natural beaver dams and/or BDA restoration structures to influence longitudinal surface water temperature regimes at the scale of stream segments (i.e., 100 m to kilometers), 2) document the potential for beaver dams and BDA restoration structures to induce channel-scale (i.e., 0.1 m to 10 m) temperature heterogeneity during periods of extreme surface water temperature during summer, 3) provide insight into the mechanisms by which beaver dams and BDA structures influence stream temperature among seasons and at multiple spatial scales. Results from this study provide a comprehensive account of the influence that beaver dams and BDA structures have on stream temperatures, and provide managers with information that can be used to design and set expectations for beaver based restoration projects.

## Methods

### Study area

**Fig 1:**
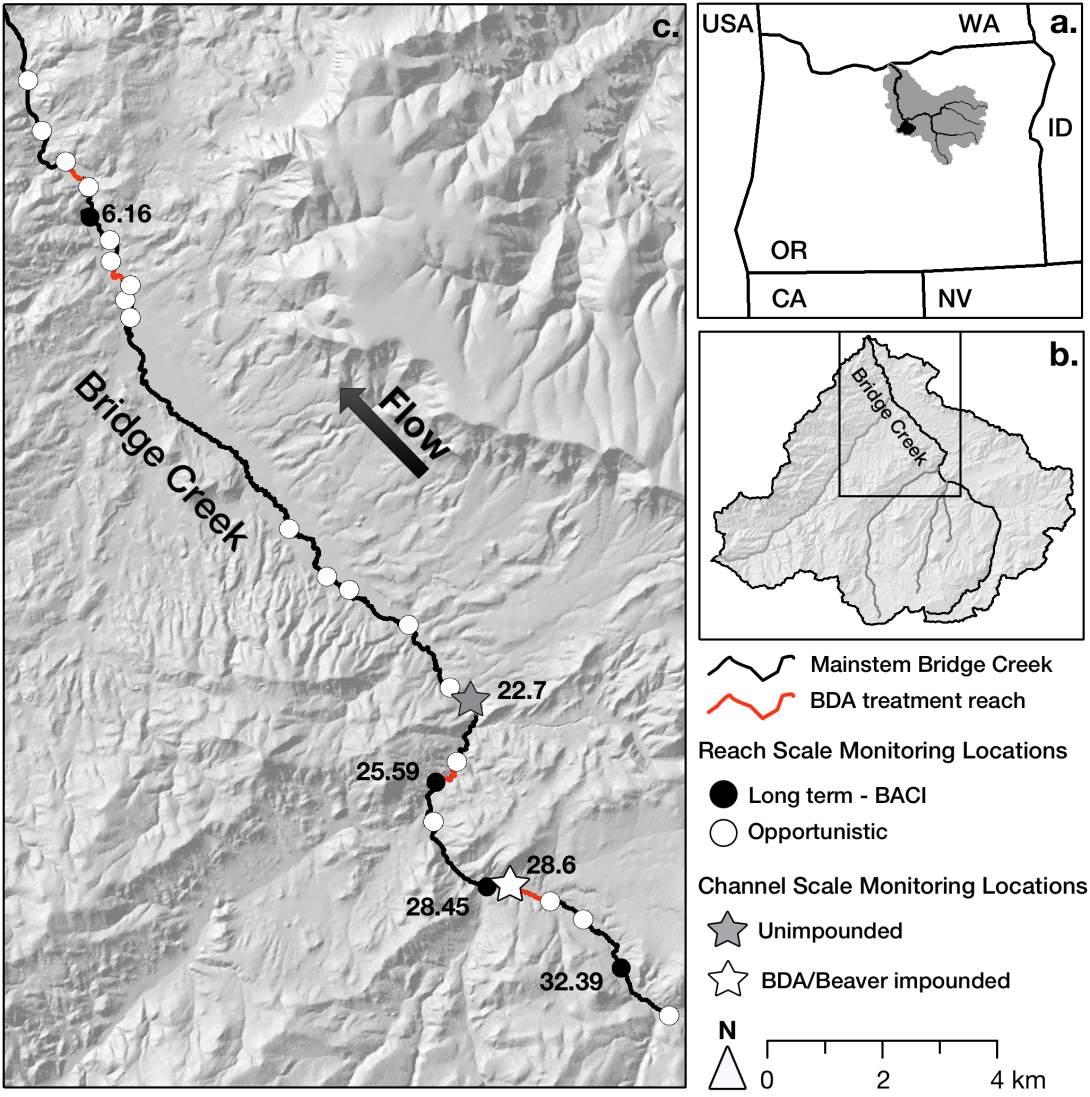
Study area map. Location of the John Day (a) and Bridge Creek (b) watersheds in OR, USA. Enlarged view (c) depicting the spatial distribution of long-term (black dots, labels are river kilometer) and opportunistic (white dots) monitoring sites on Bridge Creek (black line). Red lines show portions of Bridge Creek treated with beaver dam analog (BDA) structures. Channel scale temperature monitoring locations for the BDA/beaver impounded (white star) and unimpounded (gray star) reaches are also shown.

**Fig 2:**
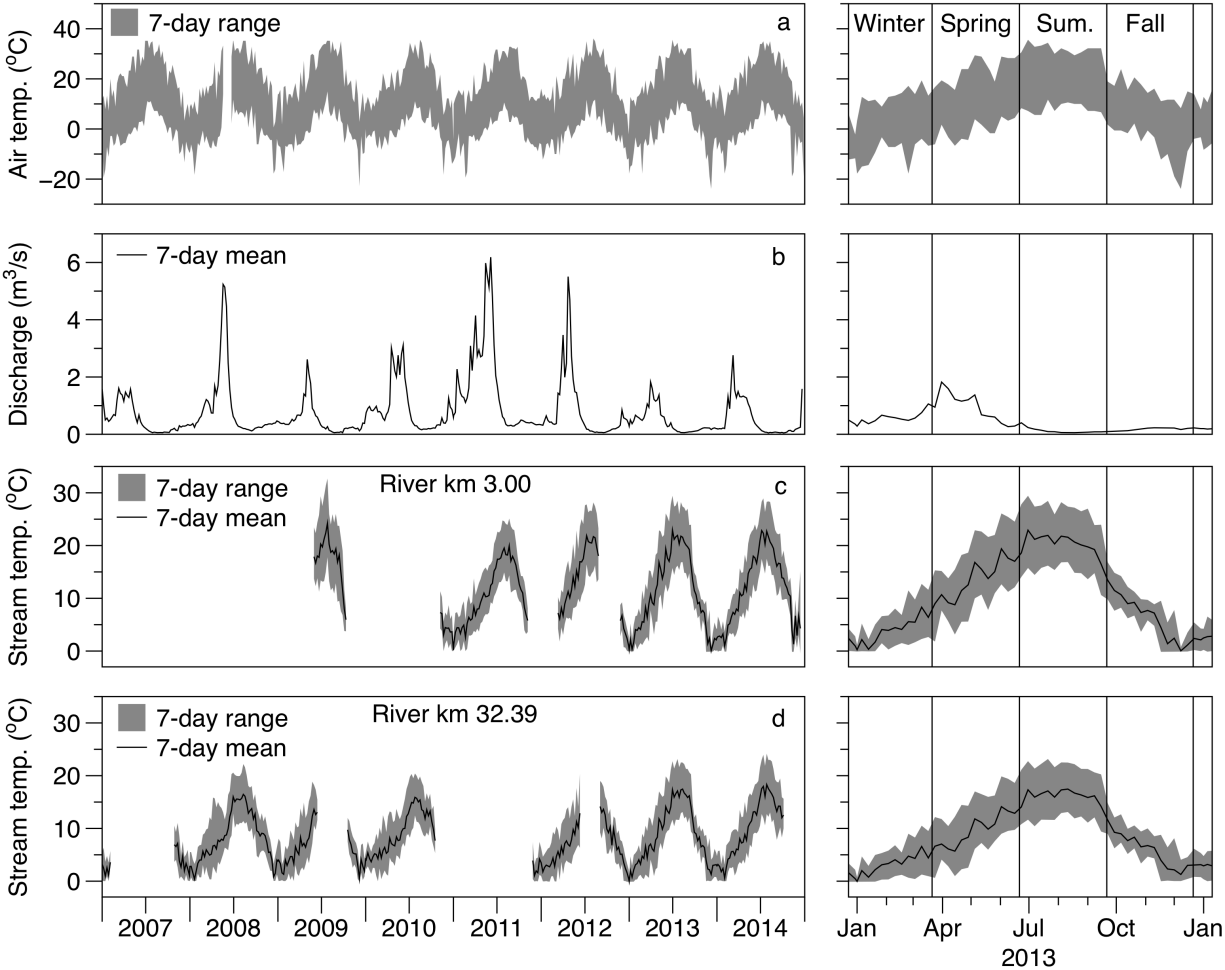
Climate, discharge, and stream temperature for the study period. Seven-day minimum and maximum air temperature range at the study area (a). Seven-day mean discharge measured near the mouth of Bridge Creek (b). Minimum, mean, and maximum daily stream temperatures for monitoring sites located in the cooler upstream (c, river km 32.39) and warmer downstream (d, river km 3.00) reaches of the study area. Gaps in temperature data indicate when a site was not operational, when a logger may have been lost during high flow, or was suspected to have been out of the wetted channel. Right panels show a more detailed view of a single year (2013) of air temperature, discharge, and stream temperatures, with vertical black lines representing astronomical seasons.

This study was conducted on the lower 34 km of Bridge Creek (44° 39’ N, 120° 15’ W), a high-desert stream flowing into the John Day River in a semi-arid portion of central Oregon (Fig 1). The Bridge Creek watershed occupies an area of 710 km^2^ and ranges between 2078 m of elevation within the Bridge Creek Wilderness to 499 m at its confluence with the John Day River. The continental climate of the region is characterized by a seasonally variable temperature regime, with air temperatures commonly exceeding 30°C during summer and routinely dropping below 0°C during winter (Fig 2, National Climate Data Center station 355638). Mean annual precipitation in the basin is roughly 30 cm, with only 3 cm occurring during summer (Table 1). Snowmelt and rain during late winter and early spring contribute to peak flow events on Bridge Creek that may exceed 6 m^3^/s, while base flow discharge may be as low as 0.05 m^3^/s for much of the dry summer season (Fig 2, USGS gauge 14046778).

**Table 1:** Study area precipitation.

Year	Seasonal precipitation (cm)				
	Winter	Spring	Summer	Fall	Annual
2007	5.9	11.6	1.5	10.1	**29.0**
2008	9.5	1.7	0.8	4.2	**16.2**
2009	5.4	11.2	5.8	8.6	**31.0**
2010	8.4	17.6	5.1	10.4	**41.5**
2011	9.9	15.9	0.9	3.2	**29.9**
2012	6.9	12.9	2.7	9.8	**32.3**
2013	6.1	10.1	3.1	9.8	**29.1**
2014	10.5	8.5	4.1	8.4	**31.6**
**Mean**	**7.8**	**11.2**	**3.0**	**8.1**	**30.1**

Seasonal precipitation (cm) for the study period (National Climate Data Center station 355638).

The headwater reaches of Bridge Creek flow through a mature forest dominated by stands of ponderosa pine (*Pinus ponderosa*), Douglas fir (*Pseudotsuga menziesii*), and Engelmann spruce (*Picea englemannii*). Within the lower elevation 34 km study area vegetation transitions to a shrub-steppe eco-type dominated by juniper (*Juniperus occidentalis*), sagebrush (*Artemesia spp*.), knapweed (*Centaurea repens*), and cheatgrass (*Bromus tectorum*). Within the study area, Bridge Creek flows through a broad valley bottom ranging between several hundred meters to over a kilometer in width. Valley soils are diverse but primarily composed of silty clay loam and coarse loamy sand with alluvial deposits of sand, gravels, and cobbles. Soil bulk densities near the active channel are approximately 1.4 g/cm, with a porosity of approximately 0.55 cm/cm [30]. Much of the channel in the lower valley is highly incised in alluvial material, and channel morphology is dictated by adjacent terraces that range in elevation from 0.5 to 3.5 m above the active channel. Active channel widths range between 4 m and 8 m with an average gradient in the range of 1% to 3%. Due to its incised nature, riparian vegetation is often reduced to a narrow (<1 m) corridor adjacent to the low-flow channel composed of various willow species (*Salix spp*.), with dogwood (*Cornus sericea*), alder (*Alnus spp*.), and cottonwood (*Populus spp*.) present in low abundance.

Stream temperatures within Bridge Creek follow a longitudinal trend that increases progressively from up to downstream, and daily maximum temperatures during summer often exceed 27°C throughout much of the study area (Fig 2). Bridge Creek provides spawning and rearing habitat for a threatened population of Middle Columbia Steelhead (*Oncorhynchus mykiss*) and moderation of high temperatures has been identified as being a critical restoration priority [31]. Bridge Creek has been the subject of a watershed scale restoration experiment that consisted of installing beaver dam analog (BDA) structures designed to accelerate the processes that contribute to channel incision recovery [27,29]. As part of the restoration experiment, steelhead populations, water quality, and channel conditions have been continually monitored throughout the lower 34 km of Bridge Creek from 2005 to 2014.

### Beaver dam analog restoration

The restoration implemented on Bridge Creek was based on the premise that the dam—building activity of beavers plays a key role in the recovery of incision—prone stream systems that exist in a state of reduced geomorphic complexity, hydrologic disconnection from groundwater and floodplains, and reduced riparian forest [27,32,33]. While a persistent population of beaver have remained present on the mainstem of Bridge Creek, lack of large—woody riparian vegetation that would serve as dam building material, and the confined condition of the channel has prevented beaver from establishing persistent dam complexes that might accelerate incision recovery [34,35]. The restoration manipulation on Bridge Creek consisted of supplementing natural beaver dams with a high density of flow resistant beaver dam analog (BDA) structures. BDAs were constructed by driving a line of 10 cm diameter posts into the stream bed to provide a stable platform on which beaver may construct dams that are more persistent and resistant to peak discharge. Posts in each line were spaced at approximately 30 to 50 cm apart, spanned the active channel, and extended onto inset floodplains. The restoration design is described in detail by Pollock [29]; in brief, BDA structures were installed within 4 reaches of the mainstem of Bridge Creek (Fig 1, river kilometer [hereafter rkm] 4.26, 7.31, 24.75, 28.45). These reaches were randomly selected from a candidate set of channel sections that featured moderate channel incision and narrow inset floodplain development that offered high restoration potential. Initial installation consisted of 80 structures installed during the Fall of 2009. Additional structures were installed in subsequent years resulting in a total of 134 structures by the summer of 2014. Permission to install BDA restoration structures on federal lands was granted by the Bureau of Land Management, the Oregon Department of State Lands, and through consultation with the Oregon Department of Fish and Wildlife and the National Marine Fisheries Service [36].

### Beaver dam distributions

We conducted a census of beaver dams throughout the lower 34 km of Bridge Creek each year of the study period over 3 to 4 days in early December. Surveys were designed to document the distribution of natural dams, and whether natural dams and BDA structures were effectively ponding and/or dispersing flow onto adjacent floodplain surfaces. In most cases, the effectiveness of BDA structures was enhanced by active maintenance by beaver, which included additions of woody vegetation and other plant material that served to increase dam crest elevations, lateral extent, and decrease dam permeability. Thus, only actively maintained BDA structures were included in subsequent analyses of beaver dam influence to stream temperature.

### Reach scale stream temperature

We monitored stream temperatures in Bridge Creek throughout the study period at 23 separate locations. Hourly temperatures were recorded using Onset^®^ temperature loggers (UTBI-001, U22-001) housed in steel cases anchored to the stream banks or directly to the stream bed. Each stream temperature monitoring site was established in un-impounded (i.e. not in a beaver pond) sections of plane-bed channel with moderate turbulence that would encourage mixing so as not to subject data loggers to channel scale temperature heterogeneity caused by local groundwater upwelling or stratification in pool or pond channel types. Loggers were always set to record temperature at the top of each hour so that measurements would be recorded nearly simultaneously regardless of location. Four long-term sites were established at the onset of the study prior to summer of 2007 (Table 2, river rkm 6.16, 25.59, 28.45, 32.39) with additional sites established opportunistically in 2008 (1), 2009 (7), 2013 (9), and 2014 (2). At full implementation in 2014 the mean distance between temperature loggers was 1.45 km (min = 0.3, max = 7.16 km). Stream temperature data were downloaded biannually, the condition of the logger recorded (i.e., out of wetted channel, buried in substrate deposition), and subjected to a quality assurance procedure prior to being included in subsequent data analyses [37].

**Table 2:** Stream temperature monitoring site locations.

Distance from mouth (km)	Distance from downstream site (km)	Operation dates		Beaver activity association
		Start date	End date	
2.05		Aug. 18, 2014	Oct. 20, 2014	
3.00	0.95	May 29, 2009	Dec. 17, 2014	
4.26	1.26	May 29, 2009	Oct. 20, 2014	
5.09	0.83	July 3, 2013	Oct. 20, 2014	
6.16	1.07	June 12, 2007	Oct. 21, 2014	Natural dams
6.81	0.65	Aug. 7, 2013	Oct. 21, 2014	
7.31	0.50	July 4, 2013	Oct. 16, 2014	
8.10	0.79	July 3, 2013	Oct. 16, 2014	
8.40	0.30	May 29, 2009	Oct. 16, 2014	
8.95	0.55	July 13, 2013	Oct. 16, 2014	
16.11	7.16	June 16, 2009	Dec. 17, 2014	
17.92	1.81	June 14, 2008	Feb. 17, 2014	
18.68	0.76	June 14, 2009	May 13, 2015	
20.50	1.82	Aug. 18, 2014	Oct. 16, 2014	
22.37	1.87	June 14, 2009	Oct. 16, 2014	
24.75	2.38	July 5, 2013	Oct. 16, 2014	
25.59	0.84	June 13, 2007	Nov. 17, 2014	Natural dams
26.56	0.97	Aug. 9, 2013	April 27, 2015	
28.45	1.89	June 13, 2007	Feb. 17, 2014	Dam analogs
29.96	1.51	July 5, 2013	Oct. 8, 2014	
30.86	0.90	June 14, 2009	Oct. 8, 2014	
32.39	1.53	June 18, 2006	Oct. 8, 2014	No dams
33.84	1.45	Aug. 9, 2013	Oct. 8, 2014	

Locations (river kilometer, distance from mouth), distance from next downstream site, and site operation dates for temperature monitoring sites on Bridge Creek. Association with upstream beaver dam or beaver dam analog (BDA) complex is also listed for the 4 long-term monitoring sites with data spanning the study period and included in the BACI (before—after—control—impact) analysis.

#### BACI analysis

**Fig 3:**
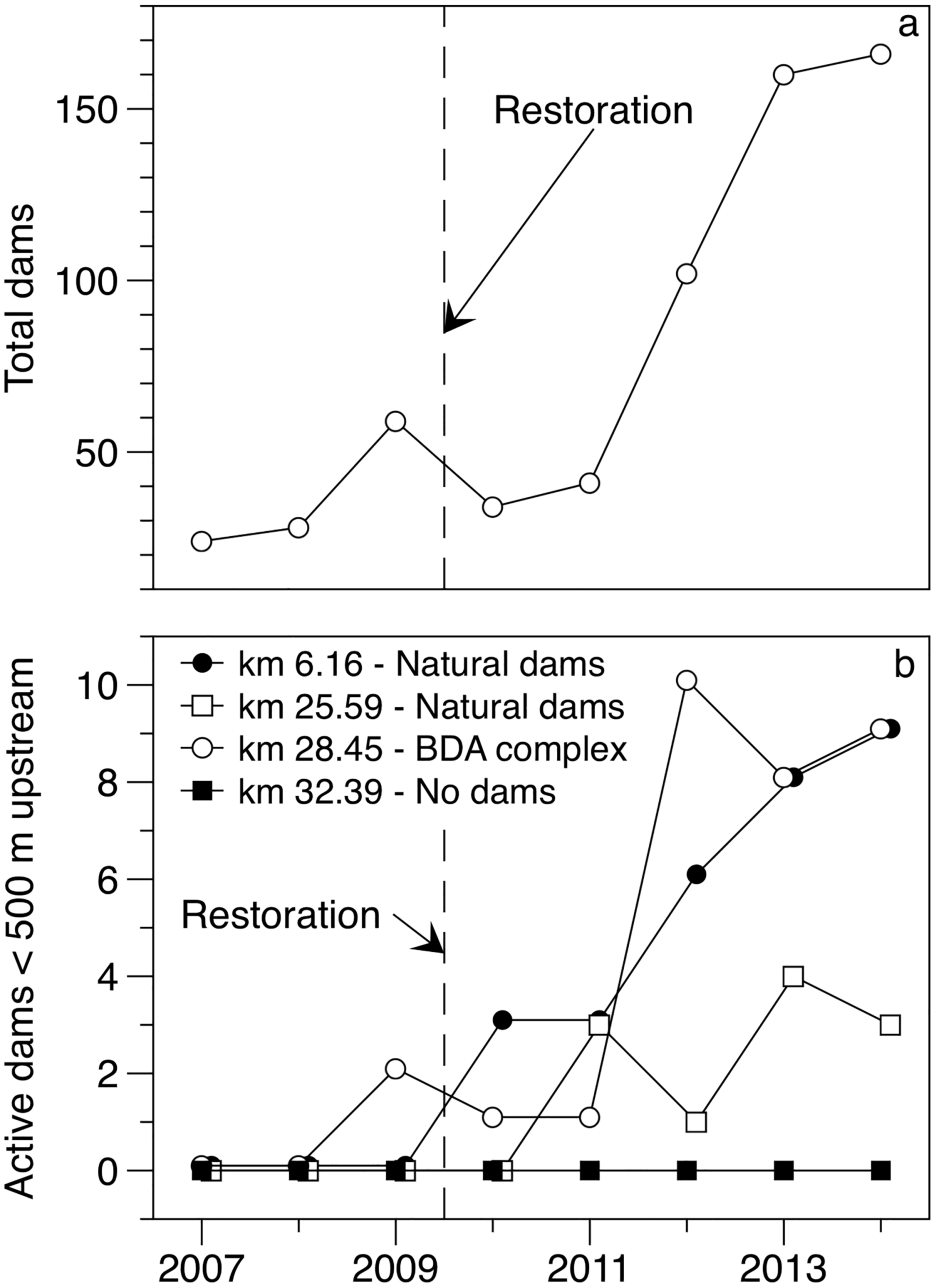
Beaver dam increase. Total number of intact natural dams and BDA structures actively maintained by beaver in the 34-km study area (a), and number of intact natural dams and BDA structures within 500 m upstream of stream temperature monitoring sites that spanned the entirety of the study period (b). Vertical hashed line shows date of BDA structure installation.

We conducted two analyses to describe the effects of natural dams and BDAs on reach scale stream temperature. Our first analysis focused on the 4 long-term monitoring sites in which stream temperature was recorded for the duration of the study period (i.e. 2007–2014). The spatial distribution and timing of BDA installation and natural beaver dam expansion upstream of these sites resulted in a set of conditions by which the influence that beaver dam complex development has on stream temperature regimes could be evaluated using a naturally occurring before—after—control—impact (BACI) [38–40] experimental design (Table 2). This design is represented by the beginning (2007–2010) and latter (2011–2014) halves of the study period, over the course of which the number of dams upstream of 3 of the 4 stream temperature monitoring sites increased by several-fold (Fig 3). Increases in dam abundance consisted solely of natural dams at two sites (rkm 6.16 and 25.59), and as the combination of natural dams and BDA structures actively maintained by beaver at the third site (rkm 28.45). The fourth long-term monitoring site (rkm 32.39), being upstream of all beaver activity and BDA treatment locations, emerged as a non-beaver influenced control. We characterized beaver activity by quantifying the number of dams and actively maintained BDA structures within 500 m upstream of each stream temperature monitoring location for each year of the study period. As an additional characterization of beaver dam activity, we quantified the change in surface area at each of the 4 locations using high-resolution aerial imagery captured during summer flows prior to proliferation of beaver dams in 2005 and following dam proliferation in 2013 [28]. Like our quantification of beaver dams, we measured wetted channel area within 500 m upstream of each location for each imagery acquisition using ArcGIS^®^.

To assess the impacts of beaver dam increases on stream temperature we calculated the difference in the minimum, mean, and maximum temperature measured at the beaver affected sites (rkm 6.16, 25.59, 28.45) and the non-beaver affected control site (rkm 32.39) for each day of the study period. The relative differences provided a measure of stream temperature that was normalized with respect to annual variation in climatic conditions that also influence stream temperature, thereby isolating the response due to the impact of beaver dam complex development alone. In accordance with the BACI design, we used one-way ANOVA to test whether the relative difference between the beaver affected sites and control site were different during periods of low (2007–2010) and high (2011–2014) dam abundance. Separate ANOVA models were used to evaluate the relative difference in daily minimum, mean, and maximum stream temperature for each season (i.e. winter, spring, summer, fall).

#### Longitudinal analysis

The second analysis focused on longitudinal trends in daily stream temperature (Fig 1, Table 2). Longitudinal stream temperature change, defined as the increase or decrease in temperature from up to downstream, was calculated by subtracting the daily minimum, mean, or maximum temperature recorded at an upstream site from the next downstream site. Thus, positive values indicate increasing and negative values indicate cooling stream temperature from up to downstream. As a measure of beaver dam influence, we calculated annual dam density (dams/km) by dividing the number of intact natural dams and BDA structures observed each year by the distance between each stream temperature site. The influence that beaver dams have on longitudinal stream temperature change was evaluated using linear regression models between annual dam density and the mean of daily longitudinal temperature change between each site. Separate regression models were run for minimum, mean, and maximum longitudinal stream temperature change for each season (i.e., winter, spring, summer, fall).

### Channel scale stream temperature

In addition to investigating reach scale stream temperature, we evaluated the potential for beaver dams to increase the distribution of groundwater upwelling zones that contribute to channel scale stream temperature heterogeneity during summer ambient air temperature extremes. To do this, we monitored near-bed stream temperatures at a high-density of locations within an active beaver complex (rkm 28.6) and within an unimpounded reach (rkm 22.7) with plane-bed channel morphology (Fig 1). The beaver complex reach contained 3 intact beaver dams during the temperature monitoring period which spanned 3 days between 22 July and 24 July, 2015. Within the complex, stream temperature was monitored at 22 locations distributed throughout 47 m of multithreaded channels and ponds, with the spacing between loggers ranging between 1.3 and 6.2 m. Temperature was monitored at 10 locations within the 19 m unimpounded reach between 5 July and 7 July 2015, with the spacing between loggers ranging between 0.5 and 3.6 m. Temperature monitoring locations were chosen opportunistically to encompass a variety of orientations to beaver dams and represent the range of water depths present in each channel type, which ranged between 0.14–0.64 m and 0.07–0.25 m in the beaver complex and unimpounded reach, respectively. Each logger (Onset^®^ UTBI-001) was set to record stream temperature at the top of each hour, and loggers were anchored in the channel on a PVC pipe so that they were suspended approximately 3 cm from the streambed. We evaluated the presence of groundwater upwelling by plotting hourly stream temperature data measured at each location, and calculating the range in minimum, mean, and maximum stream temperature among monitoring locations within each reach.

## Results

### Beaver dam distributions

The number of intact natural beaver dams within the 34 km Bridge Creek study area increased dramatically during the study period from 24 intact dams in 2007 to 120 in 2014 (Fig 3). Beaver also actively built and maintained dams on many of the BDA structures, resulting in an additional 46 active dams within the study area by 2014 (Fig 3).

### Reach scale stream temperature

#### BACI analysis

**Fig 4:**
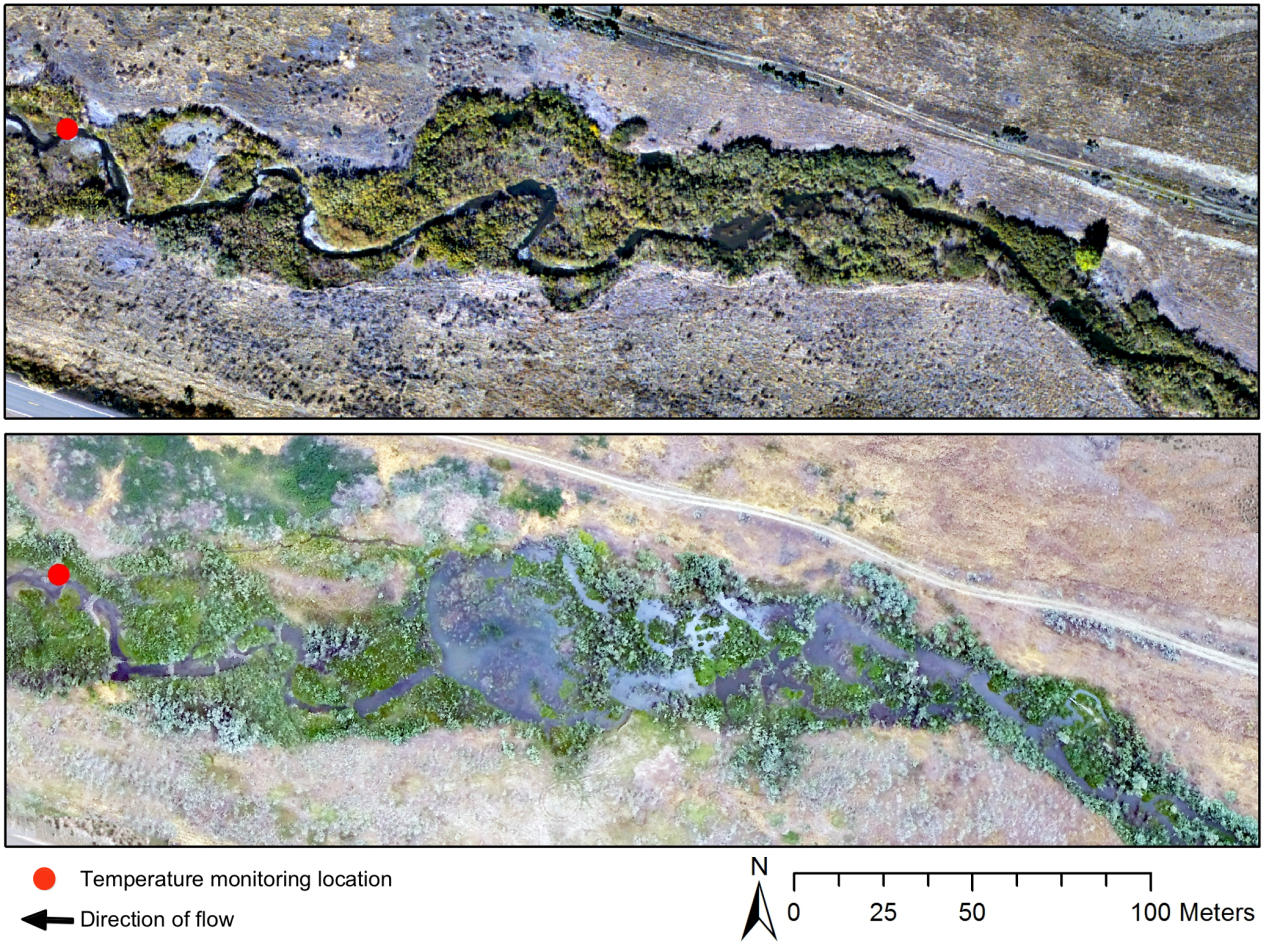
Before—After beaver colonization. Repeat aerial photography from 2005 (upper panel) and 2013 (lower panel) showing location of stream temperature monitoring site at river km 28.45 and increase in upstream pond area due to BDA implementation and proliferation of beaver activity during the study period.

Dam distributions upstream of our long-term stream temperature monitoring sites increased in a manner that was conducive to testing the influence of beaver dams per the BACI design. The number of natural dams within 500 m upstream of the site at river kilometer (rkm) 6.16 increased from 0 dams in 2007 to 9 intact dams in 2014 (Fig 3). Natural dams within 500 m upstream of the site at rkm 25.59 also increased during the study period but had only 3 intact dams in 2014. The number of intact natural dams and actively maintained dams above the site at rkm 28.45 increased from 0 dams in 2007 to 9 dams in 2014. No dams were built upstream of our control site at rkm 32.39, and aside from a small increase in the height of existing riparian vegetation the channel upstream of this site remained largely unchanged during this study. Creation of beaver dams and BDA structures resulted in a marked increase in wetted channel area and water storage upstream of the long-term stream temperature monitoring sites. Stream sections with a high amount of beaver activity increased in wetted channel area by as much as 334% during the survey period (Fig 4, Table 3). Some increase in wetted channel area (16%) was also found above the control reach that was likely due to a small difference in discharge during imagery acquisition periods.

**Table 3:** Change in wetted channel area.

River km	Beaver activity association	Wetted area (m^2^)		Δ area	% change
		2005	2013		
6.16	Natural dams	1642	7130	5488	334%
25.59	Natural dams	1286	2004	718	56%
28.45	BDA complex	1607	6149	4542	283%
32.39	Control—No dams	1167	1355	188	16%

Change in water surface area 500 m upstream of long-term stream temperature monitoring sites spanning the study period (2007–2014) and included in the BACI analysis. Water surface area was measured using high-resolution aerial photography acquired in 2005 and 2013.

**Fig 5:**
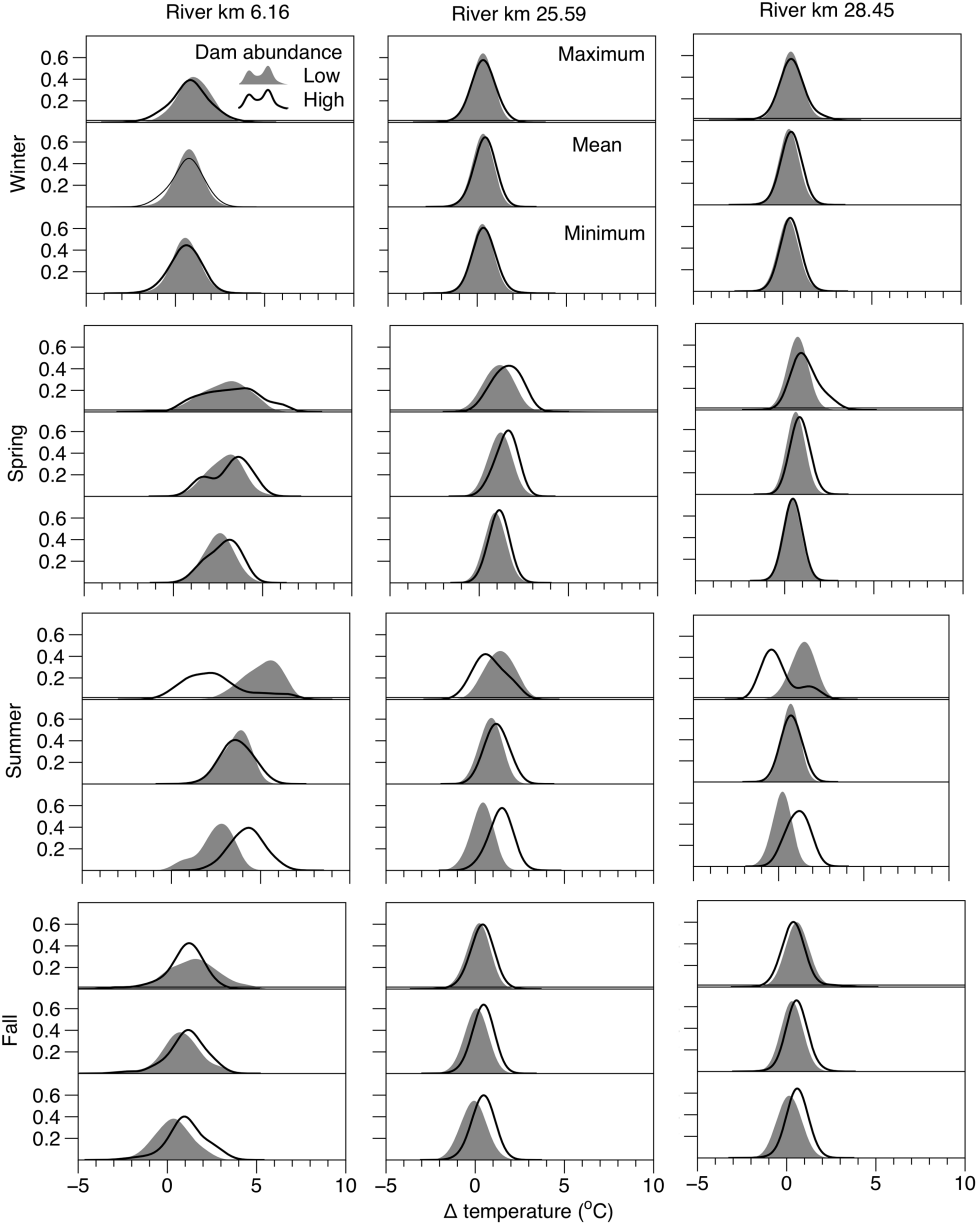
Distribution of daily stream temperature differences before and after beaver dam increase. Gaussian distribution of the seasonal difference in daily stream temperatures between the control site with no beaver dams (Rkm 32.39), and sites where dam abundance increased dramatically during the study period. Difference are expressed for each season before (grey curve) and after (black line curve) increased dam abundance for minimum, mean, and max daily stream temperature.

We observed shifts in the difference of daily stream temperature values measured at the beaver affected sites and the control site before (2007–2010) and after (2011–2014) proliferation of beaver dams (Table 4, Fig 5). At the beginning of the study daily maximum stream temperatures at rkm 6.16 were on average 5.06°C warmer than those observed at rkm 32.39 (the upstream control site with no beaver activity). Following establishment of beaver dam complexes, maximum daily stream temperature differed by only 2.5 C, representing a reduction in the mean daily relative temperature difference of roughly 2.6 C (Table 4, Fig 5). Summer minimum stream temperatures at this site were also affected by beaver complex development, which increasing by 1.76°C relative to the control site. The wetted surface area above rkm 25.59 increased by 56% following development of 4 dams within 500 m upstream of the temperature monitoring site, which also resulted in decreased maximum (0.56°C) and minimum (1.04°C) stream temperatures relative to the control. Regardless of site, deviations in relative stream temperature differences were most pronounced during summer, and daily mean was largely resistant to the influence of beaver dam complex development; however, it may be worth noting that because our unit of replication in these comparisons was on days in each season over several years, large sample sizes and high statistical power often resulted in detection of significant differences that may constitute only a fraction of a degree change in stream temperature (Table 4).

**Table 4:** BACI analysis of reach scale stream temperature.

Season	Site (river km)	Maximum		Mean		Minimum	
		Δ°C (95% CI)	*p*	Δ°C (95% CI)	*p*	Δ°C (95% CI)	*p*
Winter	6.16	-0.20 (0.18)	0.04	-0.06 (0.14)	0.42	-0.02 (0.14)	0.77
	25.59	0.02 (0.1)	0.69	0.09 (0.06)	0.00	0.10 (0.06)	0.00
	28.45	0.07 (0.09)	0.11	0.11 (0.07)	0.00	0.01 (0.08)	0.02
Spring	6.16	0.29 (0.29)	0.05	0.26 (0.20)	0.01	0.24 (0.16)	0.00
	25.59	0.25 (0.27)	0.07	0.34 (0.10)	0.00	0.21 (0.08)	0.00
	28.45	0.45 (0.09)	0.00	0.23 (0.04)	0.00	0.06 (0.03)	0.00
Summer	6.16	-2.56 (0.29)	0.00	0.04 (0.15)	0.59	1.76 (0.178)	0.00
	25.59	-0.56 (0.15)	0.00	0.34 (0.09)	0.000	1.04 (0.093)	0.00
	28.45	-1.38 (0.17)	0.00	0.05 (0.07)	0.150	0.94 (0.09)	0.00
Fall	6.16	-0.46 (0.24)	0.00	0.27 (0.20)	0.010	0.75 (0.20)	0.00
	25.59	0.21 (0.09)	0.00	0.38 (0.08)	0.000	0.53 (0.09)	0.00
	28.45	-0.19 (0.10)	0.00	0.27 (0.07)	0.000	0.44 (0.08)	0.00

ANOVA results for BACI analysis of seasonal differences in daily stream temperatures (Δ°C, 95% CI) between the upstream control site with no beaver dams (Rkm 32.39), and sites where dam abundance increased dramatically during the study period (Rkm 6.16, 25.59, 28.45). Differences are expressed for each season for maximum, mean, and minimum daily temperatures. Seasonal stream temperatures were all warmer in the beaver impacted sites prior to dam development, thus positive differences in the mean relative difference indicate increased and negative differences indicate decreased temperatures.

#### Longitudinal analysis

**Fig 6:**
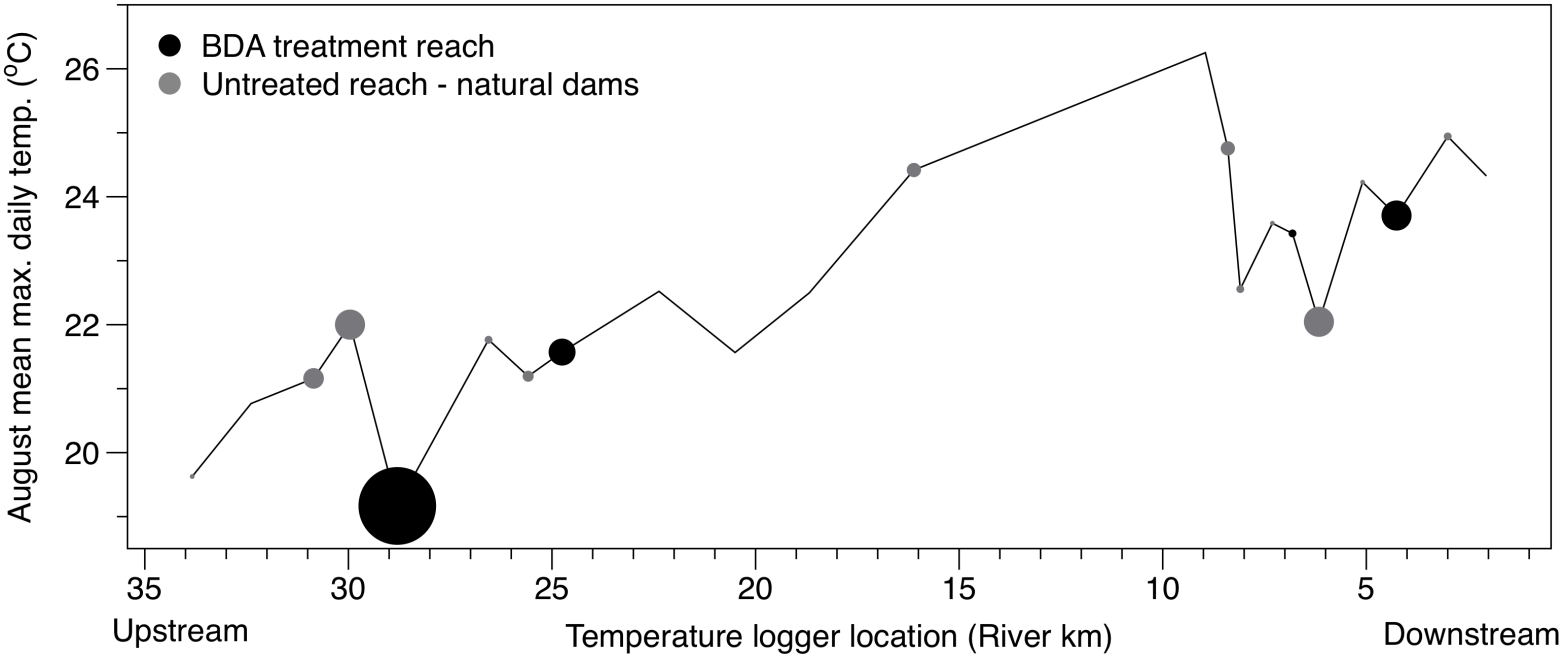
Longitudinal profile of summer maximum temperatures. Longitudinal temperature regime on Bridge Creek as the mean of maximum daily temperature observed during August 2014 among 21 monitoring locations. Points at the downstream end of each segment are sized to indicate the number of intact dams and/or beaver maintained BDA structures between monitoring locations. Black dots denote monitoring locations immediately downstream of BDA restoration reaches.

**Fig 7:**
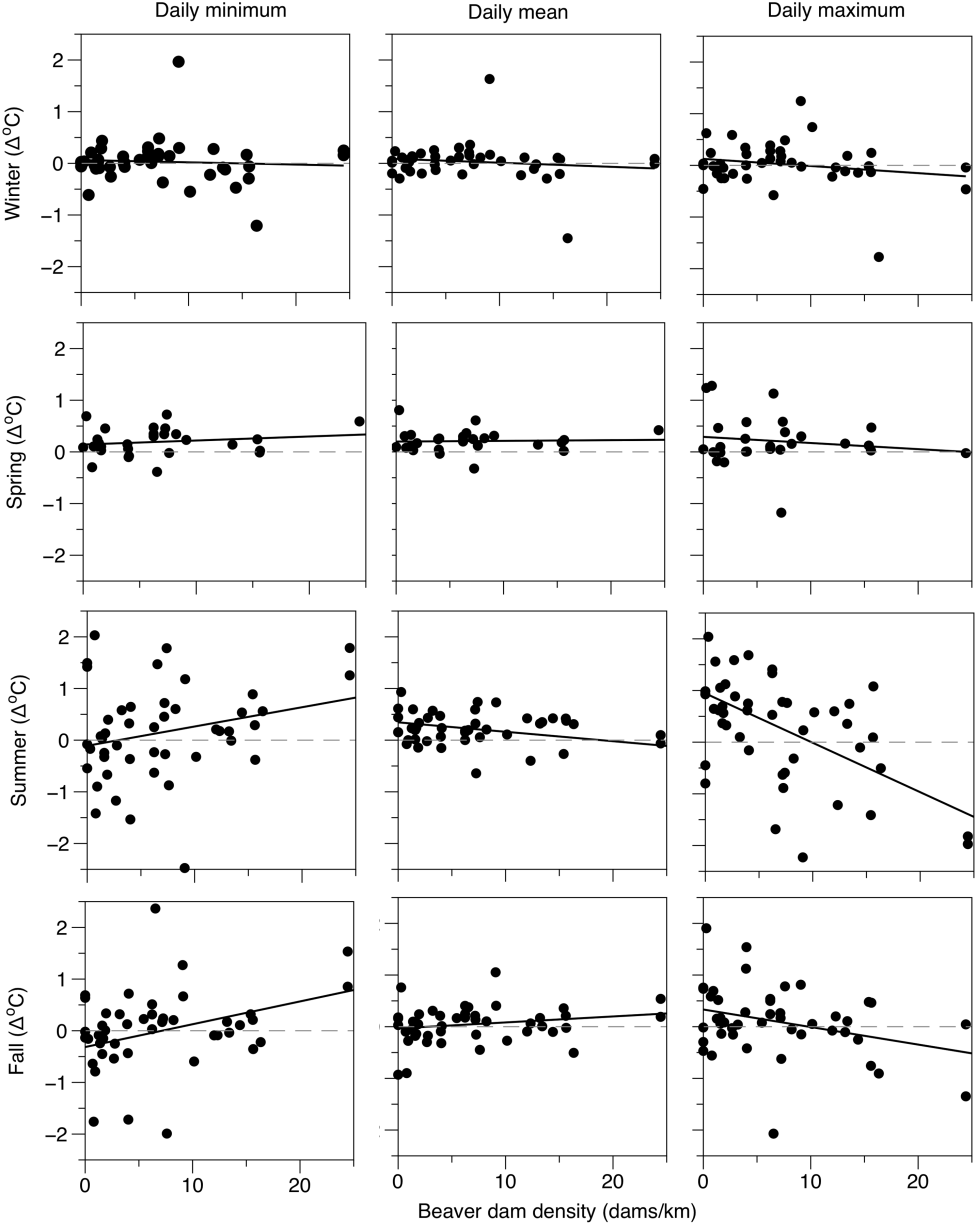
Regression analysis of longitudinal temperature change. Linear regressions showing the seasonal relationship between the mean daily longitudinal change in minimum, mean, and maximum temperature and the density of intact beaver dams (dams/km) between each temperature monitoring site. Grey hashed line at 0 provided as a reference for no longitudinal temperature change. Positive regression lines indicate increasing and negative lines indicate decreasing temperatures from up to downstream.

Analysis of longitudinal temperature patterns revealed several associations between beaver dams and stream temperature that were consistent with our BACI analysis, and suggest beaver dams can alter longitudinal temperature profiles (Table 5, Figs 6 and 7). Dam density had the strongest relationship with longitudinal stream temperature change during summer, and was most influential to minimum and maximum daily temperature. Dam density explained a significant portion of the variation in maximum longitudinal temperature change during summer (R^2^ = 0.29), and the negative slope of the relationship (-0.096) indicated that maximum summer stream temperatures are reduced in stream sections featuring a high density of beaver dams (Fig 7). For most seasons, dam density explained little of the variation in minimum, mean, or maximum longitudinal stream temperature change, and regression coefficients were often near 0 (Table 5, Fig 7). A positive slope coefficient (0.037) suggested that minimum stream temperatures may increase during summer in locations with abundant beaver dams. A weak but significant relationship between dam density and increasing minimum and decreasing maximum stream temperatures was also present during fall.

**Table 5:** Regression analysis of longitudinal temperature change.

Season	Measure	Slope (se)	Intercept	*R*^2^	df	*p*
Winter	Min	-0.004 (0.01)	0.064	0.004	41	0.68
	Mean	-0.008 (0.01)	0.090	0.017	41	0.40
	Max	-0.014 (0.01)	0.126	0.040	41	0.19
Spring	Min	0.008 (0.01)	0.140	0.020	26	0.38
	Mean	0.001 (0.01)	0.190	0.002	26	0.00
	Max	-0.012 (0.01)	0.290	0.020	26	0.45
Summer	Min	0.037 (0.02)	-0.108	0.065	44	0.09
	Mean	-0.018 (0.01)	0.340	0.033	44	0.23
	Max	-0.096 (0.02)	0.944	0.290	44	0.00
Fall	Min	0.044 (0.01)	-0.310	0.100	45	0.02
	Mean	0.011 (0.01)	-0.032	0.040	45	0.16
	Max	-0.033 (0.01)	0.330	0.100	45	0.03

Slope (se), intercept, coefficient of determination (*R*^*2*^), degrees of freedom (df), and probability of model significance (*p*) for linear regression models between the longitudinal difference in daily minimum, mean, and maximum stream temperature (°C) and annual dam density (dams/km) between monitoring sites. Slope coefficients greater than 0 indicate increases and less than 0 indicate longitudinal temperature decreases.

### Channel scale stream temperature

**Fig 8:**
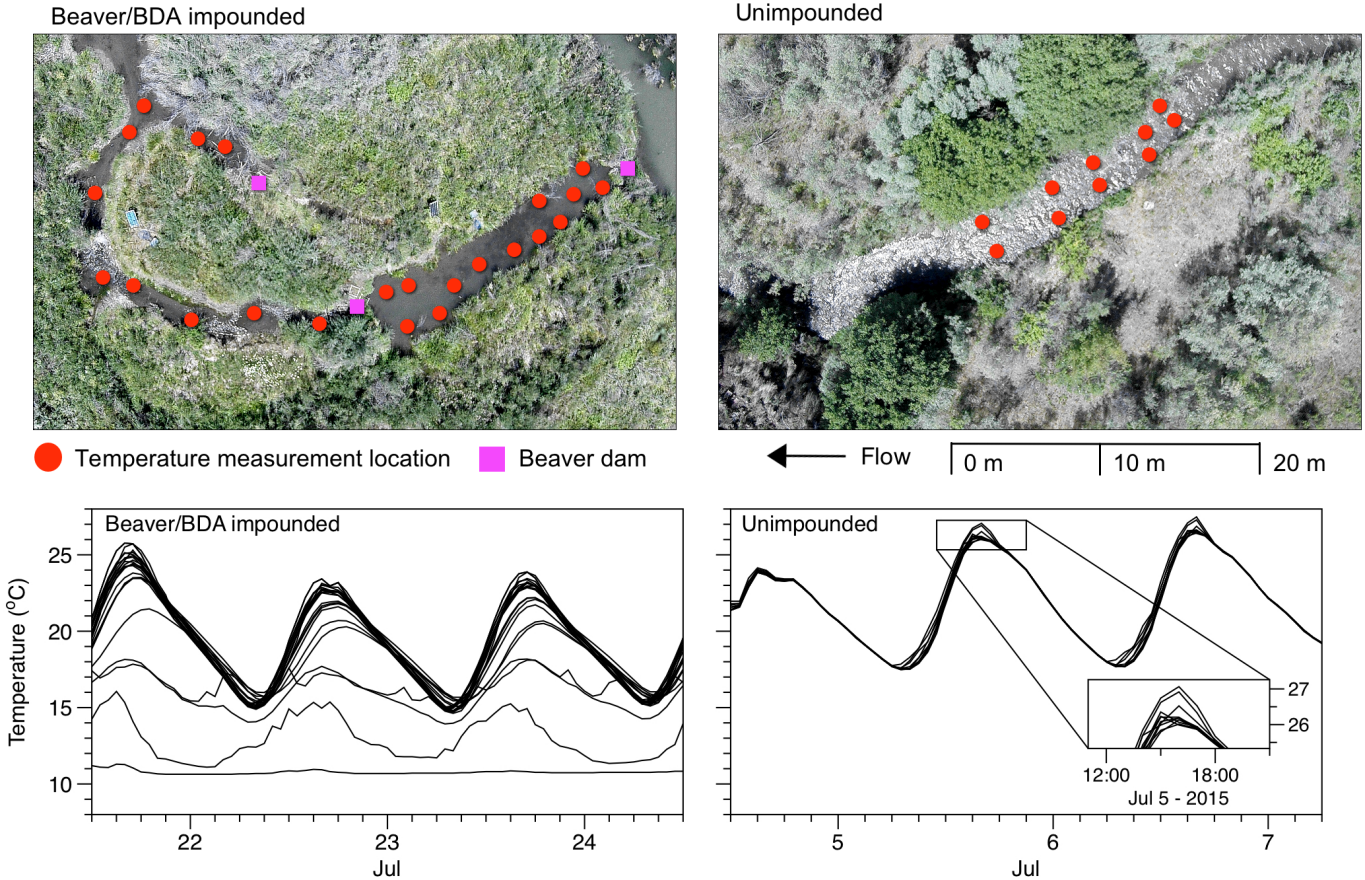
Channel scale stream temperature heterogeneity. High-density temperature monitoring locations (upper panels) and hourly stream temperature (lower panels) within the beaver dam/BDA impounded reach (left panels– 22 locations) and the unimpounded reach (right panels– 10 locations). Inset box shows narrow range of stream temperatures observed within the unimpounded reach due to lack of surface water—groundwater exchange.

The spatial distribution of near-bed stream temperatures was more diverse within the beaver complex than that of the unimpounded plane-bed reach (Table 6, Fig 8). Daily maximum temperatures occupied a range of 13.32°C (min = 11.04, max = 24.36) among the 22 stream temperature monitoring locations within the beaver dam complex. Mean and minimum daily stream temperatures also differed substantially among channel locations within the dam complex with ranges of 8.54 and 5.21°C, respectively. The magnitude of the diel stream temperature cycle was also reduced at several locations within the dam complex. In contrast, near-bed temperatures within the unimpounded reach were highly consistent among the 10 monitoring locations (Table 6, Fig 8). Minimum daily temperatures within the unimpounded reach occupied a range of only 0.21°C, and maximum temperatures were within 0.8°C among stream temperature monitoring locations.

**Table 6:** Channel scale temperature variation.

Site type	Number of locations	Daily stream temperature metrics (°C)		
		Minimum	Mean	Maximum
Beaver complex	22	10.69–15.90, 5.21	10.74–19.28, 8.54	11.04–24.36, 13.32
Unimpounded	10	18.12–18.33, 0.21	21.81–22.21, 0.40	25.42–26.23, 0.8

Mean minimum, maximum, and range (min.-max., range) for daily minimum, mean, and maximum stream temperature measurements taken during July 2015 within a beaver complex and an unimpounded stream reach.

## Discussion

Strong spatial and temporal contrasts within an experimental study design allowed us to detect deviations in stream temperature regimes that can be attributed to development of beaver dam complexes. In addition, conducting our observations and analyses at different spatial scales allowed us to identify several pathways by which beaver activity may influence stream temperature dynamics. These pathways include reach scale buffering of daily temperature extremes that result in longitudinal stream temperature discontinuities (Fig 6), and enhanced surface to groundwater connectivity causing channel scale temperature complexity (Fig 8). While the perception that beaver may contribute to increased summer stream temperatures is widespread [18,41], our observations demonstrate that beaver pond creation has the potential to alter stream temperature regimes to the benefit of temperature sensitive stream biota such as the population of steelhead that reside within our study area. In addition, our results may be one of the first to report the thermal effects of employing restoration techniques intended to encourage establishment of persistent beaver complexes in channel sections in need of a restoration intervention. As thermal degradation of freshwater environments increase in degree and extent, our results provide information that can be used to anticipate the thermal impacts that beaver dams have on stream temperatures, and provide the context for determining when beaver assisted or BDA mediated restoration may be appropriate and effective.

### Mechanisms of stream temperature alteration by beaver

One of our primary goals in this study was to identify beaver mediated mechanisms that may alter stream temperature dynamics. Our results identified two distinct ways by which stream temperature may be affected by beaver dams: a moderation of summer temperature extrema at the reach scale, and increased channel scale temperature heterogeneity. Closer consideration of these responses can serve to elucidate the mechanisms by which beaver activity modifies stream temperature regimes.

**Fig 9:**
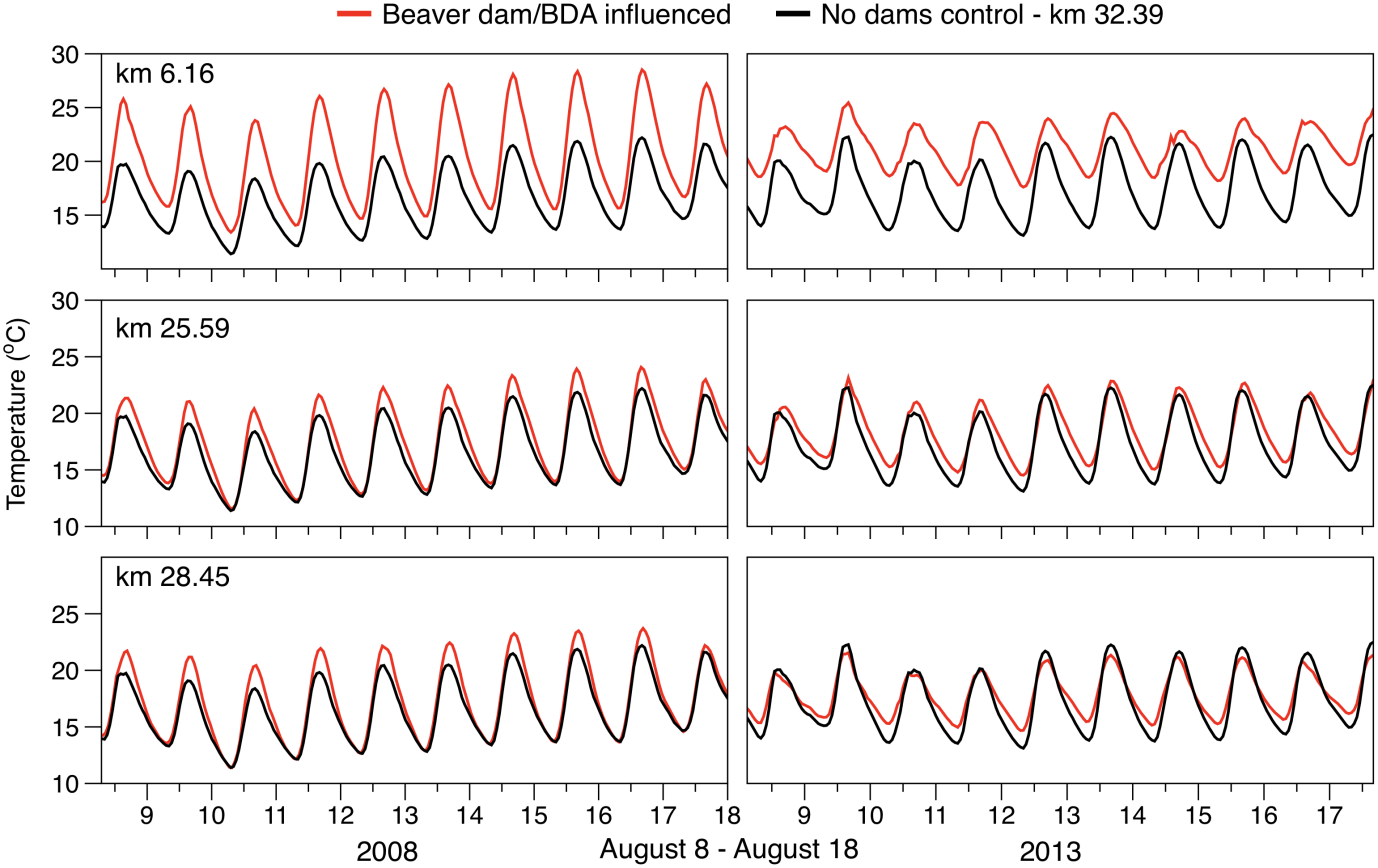
Beaver dam induced daily temperature buffering. Daily temperature regime measured within a beaver influenced site (red line) and the non-beaver influenced control site (black line) over a 10-day period in mid-summer (August 8 –August 17) for a year prior to (2008, left panels) and following (2013, right panels) BDA implementation and natural dam proliferation. Demonstrating buffering of diel temperature cycle (i.e., reduced daily range) in beaver affected sites relative to the control.

Our reach scale analyses showed a pronounced buffering of diel stream temperature cycles downstream of beaver impounded reaches during summer baseflow periods. The buffering effect of beaver dams was apparent in both our BACI analysis of beaver complex development (Table 4, Fig 5), as well as our regression analysis of longitudinal changes in daily stream temperature (Table 5, Fig 6). This response manifested as increased minimum and decreased maximum daily temperature, with no change in the daily mean. Buffered diel stream temperatures are often associated with groundwater upwelling which may exhibit a more consistent diel profile [6,42]. For example, Arrigoni [43] showed substantial buffering of diel temperature cycles (2 to 6°C) in spring channels dominated by phreatic discharge relative to that of main-channel surface flow. On small streams such as Bridge Creek, a high rate and distribution of groundwater discharge may be sufficient to induce widespread buffering of stream temperature regimes at spatial scales consistent with our observations. For example, Johnson [44] found that surface water temperatures in a small stream became strongly buffered within alluvial reaches featuring abundant hyporheic flow exchange. Beaver dam creation and dam complex development often increases groundwater exchange because of increased groundwater storage and deposition of alluvial material behind dams [20,30,42]. Lacking a before-after study design (i.e., monitoring before and after establishment of beaver dams), our comparison of near—bed temperature distributions may not be sufficient to infer that beaver dam establishment directly influences the rate and distribution of groundwater exchange. However, lack of correspondence between the diel temperature response we observed at the reach scale (Fig 9) and near—bed temperature profiles measured in proximity to groundwater upwelling zones in a beaver dam complex (Fig 8) suggest an alternative beaver-induced mechanism may be responsible for buffered diel temperature cycles.

Within the beaver complex, a single monitoring location likely heavily influenced by groundwater exhibited a nearly constant temperature of 11°C. Several other locations appeared to be influenced by cool groundwater upwelling to a lesser degree, but also followed a diel temperature cycle that would suggest mixing with surface water was also influencing the temperature profile in the immediate vicinity (Fig 8). At these locations, groundwater mixing with surface water produced a diel temperature profile that was buffered (reduced daily range) and cooled (reduced daily mean) relative to the surface water profiles not influenced by beaver dams. Based on this interaction, if increased groundwater upwelling was driving the reach scale response of surface water temperatures to beaver dams we would have expected to see an overall cooling of the diel temperature profile in addition to the compressed diel range observed.

An alternative mechanism leading to reach scale buffering of summer surface water temperatures may be solely dependent on the increased volume of surface water stored behind ponds relative to shallow unimpounded stream reaches. During summer, the dramatic diel temperature cycles on Bridge Creek are likely a product of radiant heat additions occurring during daylight, and atmospheric losses of heat via convection, conduction, and advection at night. As surface water volume is increased due to storage behind dams, the time necessary to reach extreme heat energy concentrations per these processes would also increase. This phenomenon would result in the compressed (i.e. decreased maximum, increased minimum) diel temperature cycle we observed downstream of reaches with a greater density of pond forming dams.

Stream channel shading by riparian vegetation is also thought to be an important determinant of surface water temperature regimes on smaller streams such as Bridge Creek [45]. For example, shading experiments on small streams in which solar radiation was completely blocked from surface water was shown to substantially reduce daily maximum temperatures during summer [44]. Based on our observations, it is unlikely that riparian vegetation was the primary factor driving the summer temperature response we observed. Proliferation of beaver dams on Bridge Creek generally coincided with a drastic increase in water surface area due to dam and pond creation (Table 3, Fig 4), followed shortly by a reduction of shade—providing riparian vegetation due to increased foraging pressure by beaver. While these impacts are often cited as a negative outcome of beaver colonization [17], our data suggests that any increase in radiant heating of surface water may have been offset by the buffering effect of increased surface water storage. This interaction is clear in our analysis of longitudinal temperature change, which showed a strong trend for decreasing summer maximum temperature downstream of areas with a high density of active beaver dams (Table 5, Figs 6 and 7). Additionally, the greatest longitudinal increase in summer maximum temperatures we observed occurred downstream of, and during time periods where no active beaver dams or BDA structures were present (Figs 6 and 7).

### Temperature restoration of salmonid habitat

Many salmonid populations occupy streams environments that may be at or near their thermal threshold, and small deviations in temperature may be significant in determining the population productivity and/or long-term viability [7,8]. The juvenile steelhead (< 160 mm) that rear within Bridge Creek during summer are part of a population segment thought to have a high temperature threshold; however, laboratory and field studies report extreme stress, reduced condition, and reduced metabolic efficiency occurring at approximately 25°C [46,47], and a critical maximum for survival near 29°C [48]. Maximum daily temperatures within much of our study stream (< rkm 16) exceed 25°C for much of the summer and often exceed 29°C for short periods when ambient air temperatures are high (Fig 2). Thus, any increase to stream temperatures that may have occurred because of our BDA restoration actions, or due to proliferation of natural beaver dams may have been detrimental to this already threatened steelhead population. In contrast, our observations suggest that beaver induced alterations of stream hydrogeomorphic processes result in temperature modifications that provide a benefit to cold water fish populations occupying environments that may be at or near their thermal maximum.

**Fig 10:**
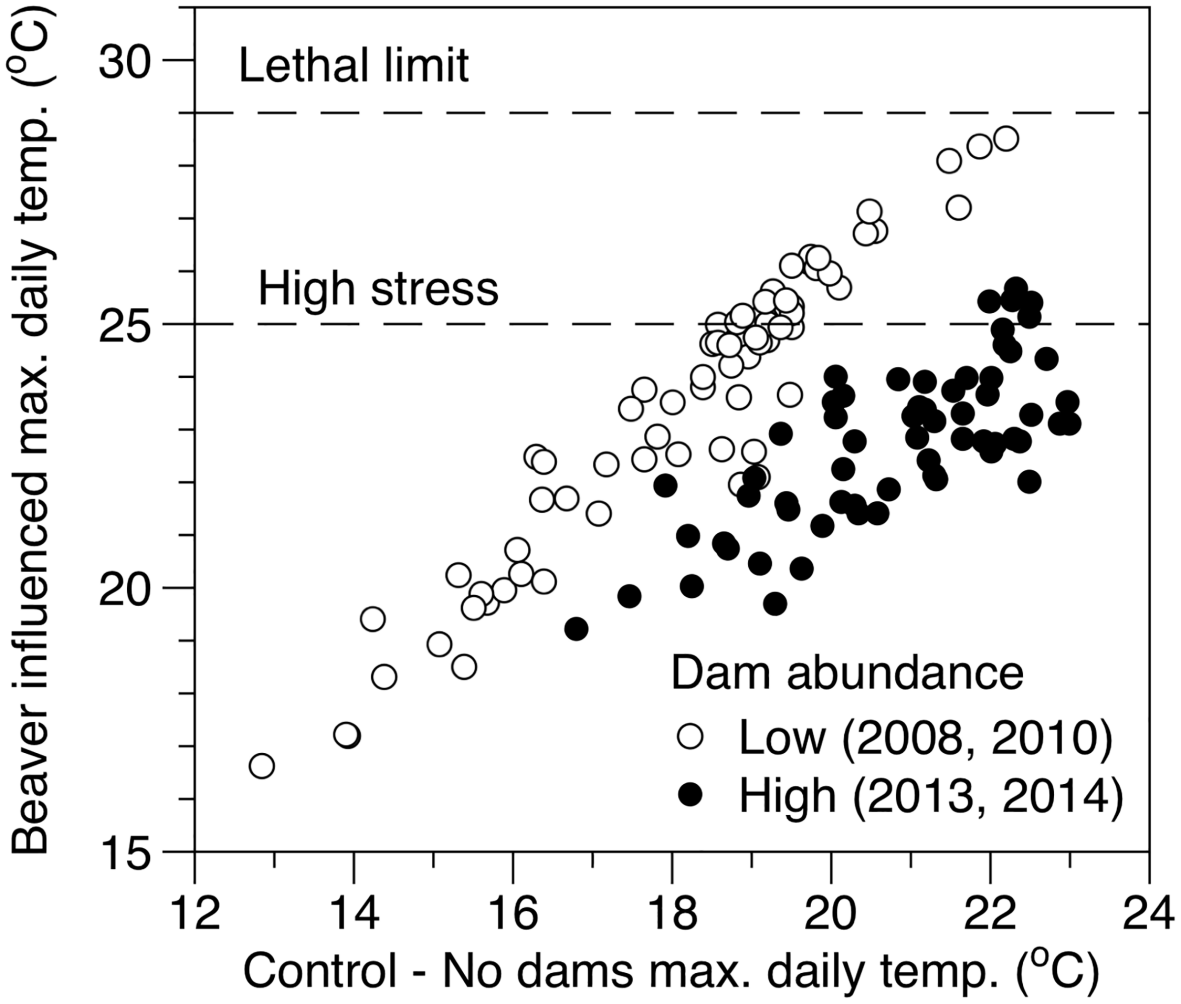
Moderation of maximum summer temperature by beaver dams. Linear relationship between August maximum daily temperatures measured below the control site with no upstream beaver activity (x-axis, rkm 32.39) and at a site (y-axis, rkm 6.16) prior to (open circles, 2008 and 2010) and following (black circles, 2013 and 2014) development of beaver dam complexes. This regression serves to demonstrate how buffering of daily temperature maxima by beavers minimizes the number of days that temperatures are above or approaching critical thresholds for juvenile steelhead (*O*.*mykiss*) during periods of extreme summer temperatures.

Although we did not observe widespread cooling of surface water temperature in our reach scale analyses, buffering of diel temperature extremes during summer has important implications for salmonid populations. For example, prior to proliferation of beaver dams (2007–2010) maximum daily temperatures at site 6.16 commonly exceeded 25°C during August when our control site with no beaver dams (rkm 32.39) was at or near 22°C (Fig 10). Following proliferation of beaver dams, daily temperatures rarely exceeded the 25°C mark despite the control site experiencing a similar annual temperature regime. In this case, establishment of a persistent beaver complex resulted in an alteration to the summer temperature regime that would reduce the thermal stress experienced by rearing juvenile salmonids. In addition, our reach scale temperature monitoring locations were often several hundred meters downstream of any beaver dam building activity, indicating that buffering of surface water temperatures by beaver impoundments was influencing downstream unimpounded reaches, thereby greatly increasing the availability of thermally suitable salmonid habitat.

Another benefit that beaver activity provides for salmonids is evident in our observations that suggest an increased distribution of groundwater upwelling zones within beaver complexes (Fig 8). Within our study area, these zones were greater than 10°C cooler than surface water temperatures measured in plane-bed channel types lacking beaver activity. Localized groundwater upwelling zones provide thermal refuge to juvenile and adult salmonids that is often critical to survival when ambient surface water temperatures exceed thermal thresholds [49], and the frequency and size of upwelling zones has been shown to limit salmonid population densities in some stream systems [50]. In addition, increased temperature complexity allows fish to select for and occupy optimal temperatures during extreme daily temperature swings that occur during summer, an opportunity that appears to be important to rearing salmonids in this system [28].

## Conclusion

Studies documenting the influence of beaver activity on stream temperature regimes have often been based on a limited spatial or temporal extent and have offered conflicting conclusions regarding the thermal impacts of beaver dam and pond creation [18]. These conflicting results are not surprising as beaver dams and beaver dam complexes exhibit a range of characteristics throughout their lifespan that will determine their influence on stream temperature regimes. Our intent in this study was to describe the impacts and mechanisms by which stream temperature regimes were altered due to a watershed scale perturbation consisting of beaver dam analog restoration and proliferation of beaver complexes. Our results suggest that increased dam and pond creation contributes to moderation of diel temperature cycles during periods of low surface flow by increasing water storage, and encouraging surface water—groundwater exchange. Many have argued that beaver pond development may lead to a degradation of thermal habitat for temperature sensitive stream biota such as threatened or endangered salmonids [41]; however, the impacts associated with a dramatic increase in beaver dams resulted in a temperature regime in greater correspondence with the thermal optima of the steelhead that utilize Bridge Creek. With beaver and salmonids once being much more abundant and widely distributed throughout North America [9,51], a commensalistic relationship between these organisms may be expected.
